# Depression, Nutritional Status, and Substance P/NK‐1R Biomarkers in Dysmenorrhea: A Randomized Crossover Trial

**DOI:** 10.1155/da/9538518

**Published:** 2026-08-27

**Authors:** Riffat Mehboob, Imran Shahid, Akash John, Shahzad Ahmad, Zeeshan Mehboob, Shadi Ahmed Alahmadi, Hisham Nasief, Ahmad Alwazzan, Khalid A. Khadawardi

**Affiliations:** ^1^ Lahore Medical Research Center, Lahore, Pakistan; ^2^ Rotogen Biotech, Bethesda, USA; ^3^ Department of Pharmacology and Toxicology, Faculty of Medicine, Umm Al-Qura University, Makkah, Saudi Arabia, uqu.edu.sa; ^4^ Faculty of Medicine and Health Sciences, The University of Buckingham, Buckingham, UK, buckingham.ac.uk; ^5^ Department of Pathology, Faculty of Medicine, King Abdulaziz University, Jeddah, Saudi Arabia, kau.edu.sa; ^6^ Department of Obstetrics and Gynecology, Faculty of Medicine, King Abdulaziz University, Jeddah, Saudi Arabia, kau.edu.sa; ^7^ Department of Obstetrics and Gynecology, Faculty of Medicine, Umm Al-Qura University, Makkah, Saudi Arabia, uqu.edu.sa

**Keywords:** depression, dysmenorrhea, neurokinin-1 receptor, substance P

## Abstract

**Substance P (SP) and neurokinin‐1 receptor (NK‐1R) as biomarkers of pain and stress:**

SP and NK‐1R are neuropeptide related biomarkers involved in nociceptive pathways. Dysmenorrhea is frequently associated with psychological distress and biochemical alterations; however, their interrelationship across treatment phases remains insufficiently understood.

**Objectives:**

To explore changes in psychological status (depression, anxiety, and stress), nutritional status, and SP/NK‐1R‐related biomarker levels across NSAID and dexamethasone plus aprepitant combination therapy phases in females with dysmenorrhea.

**Methods:**

This was a randomized, sequential, within‐subject repeated‐measures crossover exploratory controlled trial with a parallel healthy control group. A total of 40 females were enrolled, including 30 with primary dysmenorrhea and 10 healthy controls. Dysmenorrhea participants were assessed over three menstrual cycles across three phases: baseline (no treatment), NSAID phase (ibuprofen 400 mg three times daily for 3 days), and combination therapy phase (dexamethasone plus aprepitant for 2 days). Psychological status was assessed using DASS‐21, and nutritional status using the mini nutritional assessment (MNA). Serum SP and NK‐1R levels were measured using ELISA in a subset of participants due to sample limitations. Data were analyzed using IBM SPSS Statistics (version 22), with repeated‐measures ANOVA and independent *t*‐tests; *p*  < 0.05 was considered statistically significant.

**Results:**

Participants with dysmenorrhea were enrolled and assessed for ELISA based SP levels in blood. SP levels showed significant variation across treatment phases (*p* = 0.021), with the lowest values observed during the dexamethasone plus aprepitant phase compared to baseline and NSAID phases. NK‐1R levels did not show significant phase‐wise changes. Psychological subgroup analyses indicated greater SP variability in participants with moderate depression, stress, and anxiety, although several subgroups were small. Nutritional status showed partial associations with SP changes, with significant differences in malnourished and normal groups. Overall, biomarker changes were more pronounced than NK‐1R alterations across phases.

**Conclusion:**

This study demonstrates phase‐dependent changes in SP levels alongside psychological and nutritional variations in women with dysmenorrhea. However, absence of clinical pain outcomes and small subgroup sizes limit causal interpretation, and findings should be considered preliminary and biomarker‐focused.

## 1. Introduction

Dysmenorrhea is one of the most common gynecological conditions among women of reproductive age, characterized by painful menstrual cramps. It affects ~45%–95% of women, with varying intensities and associated symptoms [1]. It is classified into primary dysmenorrhea, where no underlying pelvic pathology is identified, and secondary dysmenorrhea, which is associated with conditions such as endometriosis or pelvic inflammatory disease [2]. Primary dysmenorrhea is more prevalent and is associated with physical discomfort, emotional disturbance, and psychological distress, significantly reducing the quality of life [3].

Dysmenorrhea is frequently accompanied by psychological distress, including symptoms of anxiety, depression, and stress. Evidence suggests that women with primary dysmenorrhea report higher levels of these psychological symptoms compared with those without menstrual pain [4]. A bidirectional relationship has been proposed between pain and psychological distress, in which psychological factors may influence pain perception through neurobiological mechanisms, including activation of the hypothalamic pituitary adrenal (HPA) axis and central sensitization [5]. These observations highlight the importance of considering psychological factors in dysmenorrhea; however, pharmacological strategies addressing both the psychological and somatic components remain limited.

Substance P (SP) and neurokinin‐1 receptor (NK‐1R) are neuropeptide‐related components involved in pain transmission, stress regulation, and inflammatory processes. Previous pilot evidence suggested that pharmacological modulation involving NK‐1R antagonism combined with dexamethasone was associated with changes in SP levels and pain‐related outcomes in dysmenorrhea, indicating the potential involvement of this pathway [6]. However, that study did not assess any psychological or nutritional parameters. Interest in NK‐1R antagonists has increased in recent years due to their potential role in pain‐ and stress‐related conditions. Preclinical evidence suggests possible analgesic, anxiolytic, and antidepressant effects of NK‐1R pathway modulation [7, 8]. However, the clinical evidence in dysmenorrhea remains limited, and the translational relevance of these mechanisms is still under investigation.

Dysmenorrhea is a multifactorial condition influenced by physical, psychological, and nutritional factors [9]. Nutritional status has been suggested to modulate the inflammatory and neuromuscular processes involved in menstrual pain. For example, magnesium may contribute to muscle relaxation and reduced uterine contractility [10], calcium has been associated with reduced menstrual pain possibly through prostaglandin‐related mechanisms [11], omega‐3 fatty acids have demonstrated anti‐inflammatory effects in clinical trials [12], and vitamin D deficiency has been linked with increased pain severity and psychological distress [13].

Although psychological and nutritional factors have been individually studied, limited evidence exists regarding their combined association with SP/NK‐1R‐related biomarker changes across pharmacological treatment phases. Furthermore, clinical data on NK‐1R‐related biomarkers in dysmenorrhea remain limited. Therefore, this study aimed to explore phase‐wise changes in psychological distress, nutritional status, and SP/NK‐1R‐related biomarker levels in women with primary dysmenorrhea across NSAID and dexamethasone plus aprepitant combination therapy phases. The study adopts a biopsychosocial framework to examine exploratory associations among these factors [14, 15].

## 2. Materials and Methods

### 2.1. Study Design and Participants

This study was a randomized, repeated‐measures (crossover), exploratory controlled trial with a parallel healthy control group (NCT06317064) conducted from March 2024 to October 2024. A total of 40 female participants were enrolled, including 30 women with moderate‐to‐severe primary dysmenorrhea and 10 age‐matched healthy controls without dysmenorrhea.

Participants with dysmenorrhea were followed over three consecutive menstrual cycles using a within‐subject repeated‐measures design to assess phase‐wise variations in biomarkers and psychosocial outcomes. The study included three phases: baseline (no treatment), NSAID phase, and combination therapy phase (dexamethasone plus aprepitant). A one‐menstrual‐cycle washout period was included between intervention phases, and the order of interventions was randomized to minimize carryover effects. Healthy controls were included as a reference group and underwent a single assessment during a comparable menstrual cycle phase. Controls were not exposed to any interventions and were not included in crossover comparisons.

#### 2.1.1. Study Phases

• Phase 1 (Baseline): Blood sampling prior to any intervention.
• Phase 2 (NSAID phase): Ibuprofen 400 mg three times daily for 3 days starting at the onset of menstrual pain. No additional analgesics were permitted. Adherence was monitored through medication diaries and follow‐up reminders.
• Phase 3 (Combination therapy phase): Dexamethasone (6 mg) plus aprepitant (80 mg) administered for two consecutive days during menstruation. This regimen was evaluated as a combined pharmacological exposure; therefore, the observed changes reflect combined drug effects rather than isolated NK‐1R antagonism.

**Figure 1:**
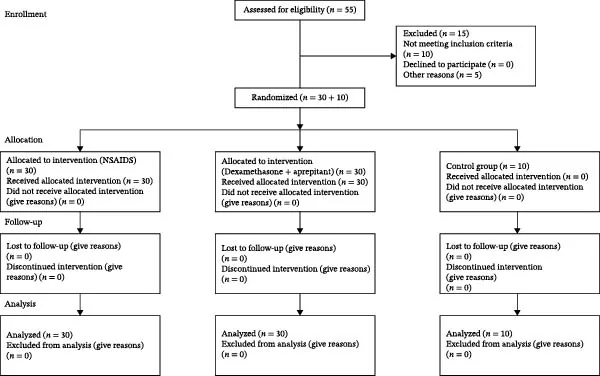
CONSORT diagram of study design for Substance P analysis.

**Figure 2:**
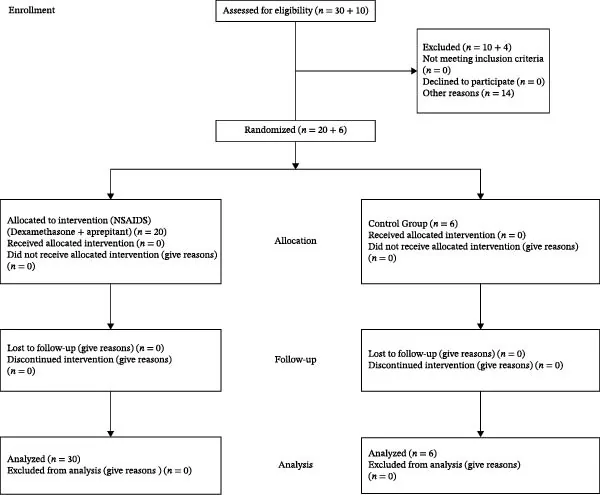
CONSORT diagram of study design for NK1R analysis.

Participant flow diagrams are presented in Figures 1 and 2.

### 2.2. Eligibility Criteria

The participants were unmarried women of reproductive age with clinically confirmed primary dysmenorrhea. Diagnosis was based on clinical history and examination after excluding secondary causes, including endometriosis, adenomyosis, uterine fibroids, pelvic inflammatory disease, and other gynecological disorders.

Pregnancy, lactation, irregular menstrual cycles, use of hormonal contraceptives (oral contraceptives or intrauterine devices), menopause, prior gynecological surgery affecting menstruation, chronic systemic illness, and use of medications affecting pain perception or inflammatory pathways were excluded.

### 2.3. Sample Size Calculation

The sample size was calculated to detect a large effect size (Cohen’s *d* = 0.8) with α = 0.05 and power = 80%, resulting in a target sample of 40 participants. However, biomarker (SP/NK‐1R) measurements were available only in a subset of participants (*n* = 20 dysmenorrhea and *n* = 6 controls) due to sample availability constraints. Therefore, biomarker and subgroup analyses were considered exploratory and underpowered, and the results should be interpreted cautiously.

### 2.4. Data Collection

A 5 mL venous blood sample was collected on the second day of menstruation at each phase (baseline, post‐NSAID, and post‐combination therapy). The serum was separated and stored under standardized conditions for biochemical analysis. A complete blood count (CBC) was also performed at each time point.

No direct clinical pain outcomes (such as pain scoring, rescue analgesic consumption, or functional disability scales) were collected. Therefore, outcome measures were limited to:

• Biomarker outcomes (SP and NK‐1R).
• Psychological outcomes (DASS‐21).
• Nutritional status (mini nutritional assessment, MNA).

### 2.5. Biomarker Measurement

Serum SP and NK‐1R‐related immunoreactive proteins were measured using enzyme‐linked immunosorbent assay (ELISA) kits (BT Lab, China) according to the manufacturer instructions. The concentrations were expressed in ng/L. All samples were analyzed in duplicate under standardized laboratory conditions, and measurements were obtained within validated calibration ranges.

### 2.6. Biomarker Assay Considerations

Although NK‐1R is a membrane‐associated receptor, the ELISA method detects circulating immunoreactive NK‐1R‐related proteins in the serum. Therefore, measured values represent relative circulating levels rather than receptor density, receptor occupancy, or functional receptor activity. Intra‐ and inter‐assay variability remained within acceptable limits (<10%–12%, respectively). NK‐1R measurements were interpreted as relative phase‐wise changes rather than indicators of receptor function or biological activity. Controls were analyzed separately and were not included in repeated‐measures phase‐wise comparisons.

### 2.7. Assessment Tools

A structured questionnaire was used to collect demographic and clinical variables, including body mass index (BMI), family history of dysmenorrhea, age at menarche, menstrual cycle characteristics, symptom duration, and sleep patterns. The psychological status was assessed using the Depression Anxiety Stress Scale (DASS‐21) [16], and nutritional status was evaluated using the MNA [17].

#### 2.7.1. DASS‐21

The psychological status was assessed using the validated DASS‐21 instrument. The scale consists of 21 items rated on a 4‐point Likert scale (0–3). Scores for depression, anxiety, and stress were calculated by summing the relevant items and multiplying by two.

Severity categories were defined as:

• Depression: normal (0–9), mild (10–13), moderate (14–20), severe (21–27), and extremely severe (≥28).
• Anxiety: normal (0–7), mild (8–9), moderate (10–14), severe (15–19), and extremely severe (≥20).
• Stress: normal (0–14), mild (15–18), moderate (19–25), severe (26–33), and extremely severe (≥34).

#### 2.7.2. MNA

Nutritional status was assessed using the validated MNA tool, which evaluates dietary intake, weight changes, mobility, and general health status.

Participants were classified as follows:

• Normal nutritional status (24–30).
• At risk of malnutrition (17–23.5):
• Malnourished (<17).

### 2.8. Informed Consent and Ethical Approval

Written informed consent was obtained from all participants after a full explanation of study objectives and procedures. Participants were informed of their right to withdraw at any time without consequences. The study was approved by the Institutional Review Board of Lahore Medical and Research Center (LMRC/2022/20/RCT003) and conducted in accordance with the Declaration of Helsinki.

### 2.9. Statistical Analysis

Data were analyzed using IBM SPSS Statistics version 22. Repeated‐measures comparisons were performed across phases. Subgroup analyses were exploratory due to the small sample sizes. A *p*‐value < 0.05 was considered statistically significant.

## 3. Results

The study included 30 women with moderate‐to‐severe primary dysmenorrhea assessed longitudinally across three repeated menstrual cycle phases (baseline, NSAID phase, and dexamethasone + aprepitant combination therapy phase), along with a small healthy control group (*n* = 10) used only for descriptive baseline comparison. The control group was not included in repeated‐measures phase‐wise analyses. Overall, most participants reported common dysmenorrhea‐associated symptoms, including cramping, fatigue, and lower back pain (90%) prior to intervention. The primary analyses focused on within‐subject phase‐wise variation in circulating SP and NK‐1R‐related biomarkers, psychological status (DASS‐21), and nutritional status (MNA).

Baseline demographic and clinical characteristics (Table 1) showed a relatively homogeneous cohort with no major variability in age, BMI, sleep duration, or menstrual cycle patterns, supporting comparability for repeated‐measures analysis.

**Table 1:** Baseline characteristics of participants (*N* = 30).

Variable	Mean ± SD
Age (years)	25.10 ± 7.604
Menarche age (years)	12.90 ± 0.7120
Age of dysmenorrhea (years)	14.90 ± 2.468
BMI (kg/m^2^)	20.47 ± 3.607
Sleep duration (hours)	6.650 ± 1.609
Length of menstrual cycle (days)	4.800 ± 0.9965

*Note:* Data are presented as mean ± SD. No missing data were recorded.

Table 1 shows that participants had relatively homogeneous baseline characteristics, including age, BMI, sleep duration, and menstrual cycle parameters. This indicates a generally comparable cohort with limited demographic variability, supporting internal consistency for repeated‐measures comparisons. As this is a descriptive table, no inferential conclusions were drawn.

Table 2 shows that SP levels varied significantly across study phases (*p* = 0.021). Lower SP levels were observed in Phase 3 (dexamethasone + aprepitant combination therapy) compared with Phase 1 (baseline) and Phase 2 (NSAID phase). This reflects a phase‐associated pattern in circulating SP; however, due to the combined pharmacological intervention, these changes cannot be attributed to isolated NK‐1R antagonism. Table 2 also shows that NK‐1R levels did not differ significantly across phases (*p* = 0.649), indicating relative stability of circulating NK‐1R‐related immunoreactivity.

**Table 2:** SP & NK‐1R levels (ng/L) between treatment and control groups.

Parameter	Control (*n* = 6)	Phase 1 (*n* = 20)	Phase 2 (*n* = 20)	Phase 3 (*n* = 20)	*p*‐Value (ANOVA)	Effect size (*ηp* ^2^)	Post‐hoc (Bonferroni)
SP	502.5 ± 123	1405 ± 1308	1361 ± 1313	778.4 ± 569.3	0.021 ^∗^	0.312	Phase 3 < Phase 1 (*p* = 0.029 ^∗^); Phase 3 vs. Control (*p* = 0.31)
	(95% CI: 375–630)	(95% CI: 808–2002)	(95% CI: 760–1962)	(95% CI: 518–1039)	—	—	—
NK‐1R	14.73 ± 7.94	12.31 ± 13.54	12.09 ± 13.37	9.12 ± 10.13	0.649	0.041	No significant pairwise differences
	(95% CI: 6.4–23.1)	(95% CI: 5.9–18.7)	(95% CI: 5.8–18.4)	(95% CI: 4.4–13.9)	—	—	—

*Note*: Values are mean ± SD with 95% confidence intervals reported in text where applicable. Repeated‐measures ANOVA was used for within‐subject comparisons. Bonferroni correction was applied for post‐hoc analyses. No imputation for missing data was performed (complete‐case analysis).

^∗^ *p*‐Value is significant (*p* < 0.05).

In addition, Table 2 shows that the healthy control group had lower mean SP levels compared with dysmenorrhea phases. However, due to the small control sample (*n* = 6), these findings are descriptive and not statistically robust for inferential comparisons.

Table 3 shows that SP levels varied across phases within the psychological subgroups. In the moderate stress category, statistically significant phase‐wise variation was observed (*p*  < 0.0001). However, several subgroup categories include very small sample sizes (including *n* ≤ 3), making these findings statistically unstable. Table 3 also shows that NK‐1R levels remained largely unchanged across phases in most psychological categories, with no consistent significant variation. Overall, Table 3 suggests a possible association between the psychological status and magnitude of SP variation across phases; however, these findings are exploratory and should not be interpreted as confirmatory due to limited statistical power.

**Table 3:** SP and NK‐1R concentrations (ng/L) across DASS‐21 stress and anxiety groups.

Group/parameter	DASS‐21 level (*f*)	Phase 1	Phase 2	Phase 3	*p*‐Value	*ηp* ^2^
DASS‐21 stress						
SP	Normal (27)	1071 ± 859.8	1113 ± 1135	775 ± 601.1	0.3217	0.042
	Mild (0)	0 ± 0	0 ± 0	0 ± 0	—	—
	Moderate (3)	4415 ± 280.5	3586 ± 51.2	808.4 ± 84.69	<0.0001 ^∗∗^	0.987
	Severe (0)	0 ± 0	0 ± 0	0 ± 0	—	—
	Extremely severe (0)	0 ± 0	0 ± 0	0 ± 0	—	—
NK‐1R	Normal (15)	16.33 ± 13.4	16.04 ± 13.26	12.04 ± 10.14	0.5718	0.039
	Mild (0)	0 ± 0	0 ± 0	0 ± 0	—	—
	Moderate (5)	0.267 ± 2.35	0.254 ± 1.98	0.356 ± 2.01	0.9966	0.001
	Severe (0)	0 ± 0	0 ± 0	0 ± 0	—	—
	Extremely severe (0)	0 ± 0	0 ± 0	0 ± 0	—	—
DASS‐21 anxiety						
SP	Normal (27)	1317 ± 963.5	1318 ± 1354	911.1 ± 702.7	0.4077	0.035
	Mild (0)	0 ± 0	0 ± 0	0 ± 0	—	—
	Moderate (3)	1537 ± 1744	1424 ± 1304	579.3 ± 138.2	0.1444	0.489
	Severe (0)	0 ± 0	0 ± 0	0 ± 0	—	—
	Extremely severe (0)	0 ± 0	0 ± 0	0 ± 0	—	—
NK‐1R	Normal (15)	16.33 ± 13.4	16.04 ± 13.26	12.04 ± 10.14	0.5718	0.039
	Mild (0)	0 ± 0	0 ± 0	0 ± 0	—	—
	Moderate (5)	0.267 ± 2.35	0.254 ± 1.98	0.356 ± 2.01	0.9966	0.001
	Severe (0)	0 ± 0	0 ± 0	0 ± 0	—	—
	Extremely severe (0)	0 ± 0	0 ± 0	0 ± 0	—	—

*Note:* Subgroup analyses with very small sample sizes (e.g., *n* ≤ 5) are considered exploratory and should be interpreted cautiously. No inferential conclusions are drawn from these subgroups.

^∗∗^ *p* < 0.01.

Table 4 shows that SP levels varied across phases in all depression categories (normal, mild, and moderate). Lower SP levels were generally observed in Phase 3 compared with earlier phases. However, Table 4 also shows that some subgroups (mild and moderate depression, *n* = 3 each) are severely underpowered. Therefore, although statistical significance is reported in some comparisons, these results should be interpreted cautiously and as exploratory only. Table 4 further shows that NK‐1R levels demonstrated minimal and inconsistent variation across phases, with no strong or consistent statistical differences.

**Table 4:** SP and NK‐1R concentrations (ng/L) across DASS‐21 depression.

DASS‐21 depression groups (*f*)		Phase 1	Phase 2	Phase 3	*p*‐Value
SP	Normal (24)	803 ± 430.1	723.2 ± 152.8	568.9 ± 96.15	0.0114 ^∗^
	Mild (3)	3211 ± 101.7	4236 ± 118	2424 ± 154.87	<0.0001 ^∗∗^
	Moderate (3)	4415 ± 280.5	3586 ± 51.2	808.4 ± 84.69	<0.0001 ^∗∗^
	Severe (0)	0 ± 0	0 ± 0	0 ± 0	—
	Extremely severe (0)	0 ± 0	0 ± 0	0 ± 0	—
NK‐1R	Normal (10)	24.3 ± 8.23	23.9 ± 8.22	17.85 ± 6.89	0.1374
	Mild (5)	0.385 ± 1.57	0.311 ± 2.21	0.425 ± 1.92	0.9955
	Moderate (5)	0.267 ± 2.35	0.254 ± 1.98	0.356 ± 2.01	0.9966
	Severe (0)	0 ± 0	0 ± 0	0 ± 0	—
	Extremely severe (0)	0 ± 0	0 ± 0	0 ± 0	—

*Note*: Mild and moderate depression subgroups (*n* = 3 each) are underpowered; therefore, results are exploratory and not intended for strong inferential conclusions.

^∗^ *p*‐Value is significant (*p* < 0.05).

^∗∗^ *p*‐Value is significant (*p* < 0.01).

Table 5 shows that SP levels varied across phases within the nutritional subgroups. Statistically significant differences were observed in some categories (normal and malnutrition groups). However, uneven and small subgroup sizes limit the robustness of these findings. Table 5 also shows that NK‐1R levels remained relatively stable across nutritional categories, with no consistent significant phase‐wise changes. Overall, Table 5 suggests possible associations between nutritional status and SP variation across phases; however, these findings are exploratory and not confirmatory.

**Table 5:** SP/NK‐1R concentrations in participants with nutritional assessments measured using MNA scale.

Neuropeptides	MNA groups (*f*)	Phase 1	Phase 2	Phase 3	*p*‐Value
SP	Malnutrition (3)	543.7 ± 29	642.2 ± 44.77	511.4 ± 21.65	0.0071 ^∗^
	Risk of malnutrition (21)	1732 ± 1450	1617 ± 1504	870.3 ± 661.1	0.0644
	Normal (6)	691.1 ± 76.22	821.5 ± 106.4	590.3 ± 102.9	0.0031 ^∗^
NK‐1R	Malnutrition (0)	0 ± 0	0 ± 0	0 ± 0	—
	Risk of malnutrition (15)	10.92 ± 15.51	10.75 ± 15.33	8.387 ± 11.71	0.8635
	Normal (5)	16.49 ± 4.87	16.12 ± 3.97	11.32 ± 7.43	0.3038

*Note:* Given unequal group sizes, subgroup comparisons were treated as exploratory. Repeated‐measures ANOVA was used for within‐group comparisons only.

^∗^ *p*‐value is significant (*p* < 0.05).

## 4. Discussion

Dysmenorrhea, characterized by painful menstrual cramps, is a common gynecological condition affecting women of reproductive age and is frequently associated with psychological distress and potential nutritional imbalances [18]. In this exploratory study, we evaluated phase‐wise variations in circulating SP and NK‐1R‐related immunoreactivity alongside psychological (DASS‐21) and nutritional (MNA) assessments in women with primary dysmenorrhea.

Across the three study phases, lower SP levels were observed during the dexamethasone plus aprepitant combination therapy phase (Phase 3) compared with baseline and NSAID phases. These observations reflect within‐cohort biomarker variability across treatment conditions. However, no clinical pain outcomes were collected; therefore, these findings cannot be interpreted as evidence of clinical improvement in dysmenorrhea. NK‐1R‐related immunoreactivity showed relatively stable patterns across all phases, with no consistent statistically significant variation.

Previous literature has highlighted the involvement of NK‐1R‐mediated pathways in stress and affective regulation. For example, NK‐1R antagonism has been explored in functional and stress‐related disorders, including its effects on emotional arousal in irritable bowel syndrome populations [19]. Meta‐analytic evidence also indicates that women with primary dysmenorrhea have higher odds of depressive symptoms compared with non‐dysmenorrheic women [20]. These findings support the broader association between dysmenorrhea and psychological distress; however, direct mechanistic evidence linking NK‐1R pathways to dysmenorrhea symptom modulation remains limited.

NSAIDs are widely used for dysmenorrhea management due to their inhibition of prostaglandin synthesis and consequent reduction in uterine contractility and pain. In the present study, biomarker and psychometric measures were described across NSAID and combination therapy phases. However, in the absence of pain scores or functional outcomes, no inference regarding comparative clinical efficacy can be made. Therefore, all observations remain limited to biomarker and psychometric variability.

Non‐pharmacological interventions have also been reported to improve the psychological outcomes in dysmenorrhea. For instance, structured psychological interventions such as stress inoculation training have been associated with improvements in pain‐related and psychological outcomes in affected populations [21]. These findings highlight the multifactorial nature of dysmenorrhea; however, comparisons with the present study should be made cautiously given the differences in study design and outcome measures.

The present study also observed descriptive variations between nutritional status categories and SP levels. Nutritional factors such as magnesium, calcium, omega‐3 fatty acids, and vitamin D have been previously implicated in menstrual health and inflammatory regulation [10–13]. However, in this study, the observed differences should be interpreted as preliminary and exploratory due to small and uneven subgroup sizes.

Importantly, Phase 3 involved dexamethasone plus aprepitant combination therapy; therefore, any observed biomarker changes cannot be attributed specifically to isolated NK‐1R antagonism. The combined pharmacological exposure limits mechanistic interpretation regarding NK‐1R‐specific effects.

Several limitations must be acknowledged. The study was conducted at a single center with a relatively small sample size. Biomarker analyses were further limited to a subset of participants due to sample availability constraints. The follow‐up duration was limited to three menstrual cycles. The psychological and nutritional assessments were self‐reported, which may introduce variability. Importantly, no direct clinical dysmenorrhea outcomes (such as pain scores, analgesic consumption, or functional disability measures) were collected; therefore, findings are restricted to exploratory biomarker and psychometric associations rather than clinical efficacy.

## 5. Conclusion

This study demonstrated phase‐wise variations in circulating SP levels and psychometric measures across baseline, NSAID, and dexamethasone plus aprepitant combination therapy phases in women with dysmenorrhea. Lower SP levels were observed during the combination therapy phase. The psychological status and nutritional categories also showed variations across phases and subgroups. No direct clinical pain outcomes were assessed, and the findings are limited to biomarker and psychometric measurements.

## Funding

The authors have nothing to report.

## Conflicts of Interest

The authors declare no conflicts of interest.

## Data Availability

The data for this research will be available upon request.
